# Meta-analysis of the potential role of extracorporeal shockwave therapy in osteonecrosis of the femoral head

**DOI:** 10.1186/s13018-018-0861-7

**Published:** 2018-07-03

**Authors:** Yangquan Hao, Hao Guo, Zhaochen Xu, Handeng Qi, Yugui Wang, Chao Lu, Jie Liu, Puwei Yuan

**Affiliations:** 1 Department of Osteonecrosis and Joint Reconstruction, Honghui Hospital Xi’an Jiao Tong University Health Science Center, No. 555 Youyi East Road, Xi’an, Shaanxi 710054 People’s Republic of China; 2 Shaanxi University of Chinese Medicine, Shiji Ave, New Economic Zone, Xi’an-Xianyang, Shaanxi 712046 People’s Republic of China

**Keywords:** Osteonecrosis of the femoral head, Extracorporeal shockwave therapy, Pain score, Harris hip score, Meta-analysis

## Abstract

**Background:**

We aimed to evaluate the role of extracorporeal shockwave therapy (ESWT) in improving osteonecrosis of the femoral head (ONFH).

**Methods:**

We searched studies focusing on the role of ESWT in ONFH using PubMed, Embase, the Cochrane Library, WanFang, VIP, and CNKI databases updated up to July 28, 2017, without language restriction. Standardized mean difference (SMD) values and 95% confidence intervals (95% CIs) were pooled to compare the pain score and Harris hip score for ESWT treatment and other treatment strategies.

**Results:**

Four articles, including 230 ONFH patients, were eligible for the meta-analysis. No significant differences were found in the pain score (SMD = − 1.0104; 95% CI − 2.3279–0.3071) and Harris hip score (SMD = 0.3717; 95% CI − 0.3125–1.0559) between the two groups before treatment. After treatment, significant differences were found between the experimental and control groups in the pain score (SMD = − 2.1148; 95% CI − 3.2332–0.9965) and Harris hip score (SMD = 2.1377; 95% CI 1.2875–2.9880). There were no significant differences in pain score before and after treatment between the two groups (SMD = − 0.7353; 95% CI − 2.1272–0.6566), but significant differences were found in the Harris hip score (SMD = 1.2969; 95% CI 0.7171–1.8767).

**Conclusion:**

For patients at an early stage, ESWT may be safe and effective for relief of pain and improvement of motor function.

## Background

Osteonecrosis of the femoral head (ONFH) is a pathological process that follows ischemic insult [1]. High morbidity occurs in both young and old worldwide [2]. In China, 8.12 million patients have been diagnosed with ONFH as of 2017 [3, 4], and the average annual number of new cases in Korea was 14,103 [5]. The occurrence of osteonecrosis is associated with various risk factors including trauma, hip surgery, corticosteroid use, alcoholism, and coagulopathy [6]. The treatment of ONFH remains a challenge, and a standardized and improved treatment strategy for ONFH is urgently needed.

“Joint-preserving” treatments, including both surgical (such as core decompression, trochanteric rotational osteotomy, and vascularized bone grafts) and conservative approaches [extracorporeal shock wave therapy (ESWT) and pulsed electromagnetic field] have been developed to prevent progression of ONFH [7, 8]. ESWT is used in physical therapy, orthopedics, urology, and cardiology, and a previous study demonstrated that the technology can successfully treat ONFH [9]. However, the efficacy of ESWT compared with other treatments remains unclear [10, 11]. For example, no significant difference in efficacy was found between ESWT and core decompression in a study by Wang et al. [10]. A study by Chen and colleagues demonstrated better outcomes with ESWT than with physical therapy [12]. Although the effect of ESWT in the treatment of ONFH has been investigated by previous researchers, there is no consistent conclusion about its efficacy when compared with other treatments.

This meta-analysis was performed to evaluate the role of ESWT in improving ONFH. Standardized mean difference (SMD) values and 95% confidence intervals (95% CIs) were pooled to compare the pain score and Harris hip score for ESWT treatment and other treatment strategies.

## Methods

### Study selection

Studies were selected using PubMed (http://www.ncbi.nlm.nih.gov/pubmed), Embase (http://www.embase.com), the Cochrane Library (http://www.cochranelibrary.com), WanFang, VIP, and CNKI databases updated to July 28, 2017, without language restriction. A combination of Medical Subject Headings (MeSH) terms and free-text keywords were used for study selection: (“ESWT” OR “Extracorporeal shock wave”) AND (“osteonecrosis” OR “Osteonecrosis” OR “femoral head necrosis” OR “ONFH” OR “Osteonecrosis of the Femoral Head” OR “avascular necrosis of femoral head” OR “necrosis of the femoral head” OR “avascular necrosis of bone” OR “Kienbock disease” OR “Aseptic necrosis of bone”).

### Selection criteria

Literature focusing on the efficacy of ESWT in patients with femoral head necrosis were included in the meta-analysis. Studies were included in the meta-analysis if they met the following criteria: (1) published Chinese or English language literature focusing on the efficacy of ESWT in patients with ONFH, in which the experimental group was treated with ESWT and the control group received a different treatment strategy; (2) reported outcomes included the pain score and Harris hip score at baseline and corresponding scores after a period of treatment; and (3) research designed as an interventional study.

Exclusion criteria were as follows: (1) incomplete data or data that could not be used for statistical analysis and (2) reviews, letters, and comments. In addition, if studies duplicated published literature or data for the same population, only the latest research with the most comprehensive information was included.

### Data extraction and quality evaluation

The authors independently extracted the following data from the included literature: the first author’s name, year of publication, study period, stage of ONFH (according to Association Research Circulation Osseous), type of study, follow-up duration, baseline characteristics of enrolled patients (e.g., sex ratio, age composition), baseline pain and Harris hip scores, and corresponding scores after treatment, sample sizes, and general demographic data.

The quality of randomized controlled trials was evaluated using Cochrane Collaboration recommendations [13]. Disagreements were resolved by discussion or by consultation with a third reviewer.

### Statistical analysis

Meta-analysis was performed using R 3.12 software (R Foundation for Statistical Computing, “meta” package, Beijing, China). The SMD values and 95% CIs were pooled to compare the pain score and Harris hip score for ESWT treatment and other treatment strategies. For *P* < 0.05 or *I*^2^ > 50%, the random effects model was used to calculate the combined effect value. Otherwise, the fixed effects model was chosen to combine data [14]. Publication bias was assessed using Egger’s method. Finally, sensitivity analysis was performed by omitting one study at a time to determine the effect on the overall SMD value.

## Results

### The general characteristics of included studies

**Fig. 1:**
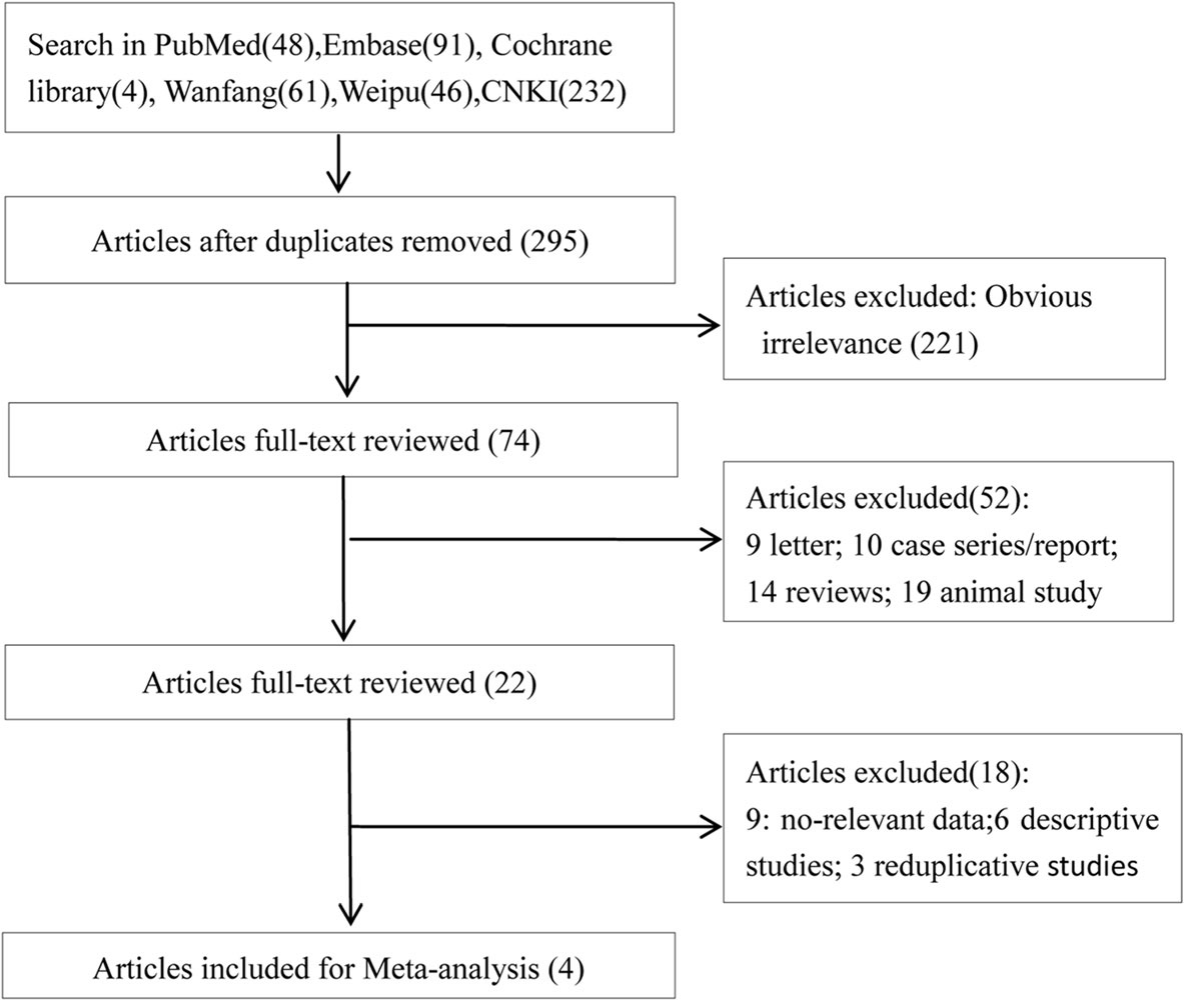
Flow chart of study selection

The flow chart used for study selection is shown in Fig. 1. Of 482 articles initially reviewed, 48 were from PubMed, 91 from Embase, 4 from the Cochrane Library, 61 from WanFang, 46 from VIP, and 232 from CNKI. After excluding duplicated literature, 295 articles were left. Then, the title and abstract were reviewed, and 221 articles obviously inconsistent with the inclusion criteria excluded. Subsequently, a total of 74 articles were fully reviewed, and 52 articles were excluded including 9 letters, 14 reviews, 10 case series/reports, and 19 animal studies. Moreover, another 18 articles including 9 articles without relevant data, 6 descriptive studies, and 3 reduplicative studies were excluded. Finally, 4 articles were included in the meta-analysis [10, 12, 14, 15].

**Fig. 2:**
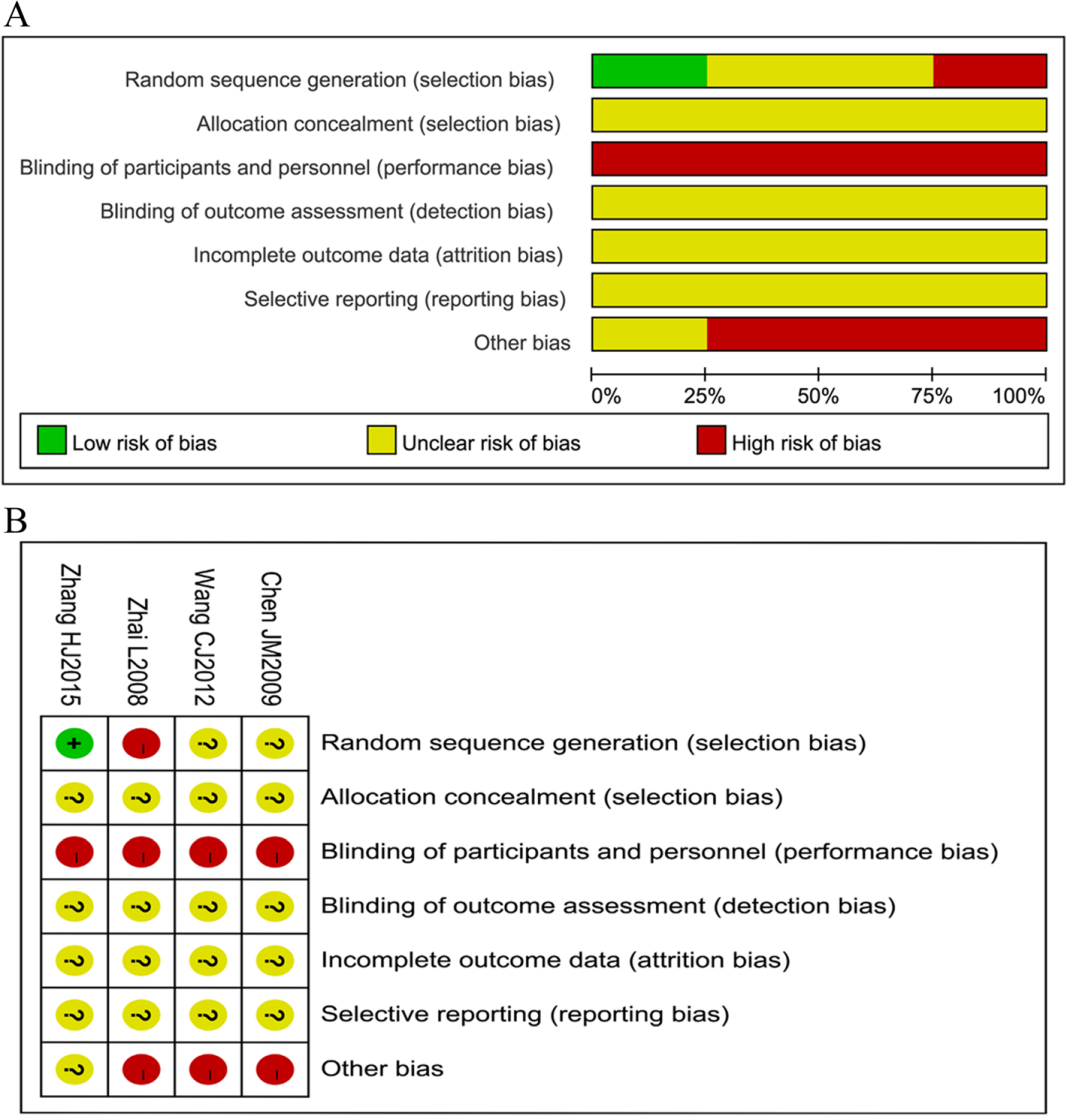
Quality assessment of the meta-analysis. **a** Risk of bias. **b** risk of bias summary

A total of 230 patients (185 men and 45 women, a significant difference) with ONFH were enrolled in this study, including 120 in the experimental group and 110 in the control group. The general characteristics of the selected literature are shown in Table 1. The publication year ranged from 2008 to 2015. Patients with ONFH were mainly in stages I–III. Only one randomized controlled study was included. No significant difference in sex or age distribution was found in individual studies. Follow-up time in three studies was more than 1 year. Figure 2 shows that the quality of the included literature was relatively poor.

**Table 1:** The baseline characteristics of included studies

Study	Year	Study year	ONFH stage	Study style	Group	Number/hips	Gender (M/F)	Age (year)	Duration (month)
Wang CJ	2012	2001–2001	Stages I, II, early III	Non-RCT	ESWT	23/29	20/3	39.8 ± 12.1	25.2 ± 3.7
					Surgical group	25/28	22/3	39.9 ± 9.3	25.8 ± 4.6
Chen JM	2009	1999.7–2006.1	Stages I–III	Non-RCT	ESWT	17/17	14/3	42.9 ± 9.3	11.3 ± 3.4
			Stages I–IV		THA	17/17	14/3	42.9 ± 9.3	14.7 ± 0.93
Zhang HJ	2015	2009.1–2012.12	Stages I–II	RCT	BMSC+ESWT	20/29	15/5	36.1 ± 6.2	24
					BMSC	20/27	14/6	35.5 ± 5.7	24
Zhai L	2008	1998.1–2007.6	Stages I–III	Non-RCT	CD + ESWT	50/50	41/9	20.9(18–25)	6.4(3–18)
					CD	58/58	45/13	20.5(18–25)	7.3(3–20)

*ESWT* extracorporeal shock wave treatment, *THA* total hip arthroplasty, *CD* core decompression, *M/F* males/females, *RCT* randomized controlled trail, *ONFH* osteonecrosis of the femoral head, *THA* total hip, *BMSC* bone marrow stem cells

### Meta-analysis of pain score and Harris hip score

**Fig. 3:**
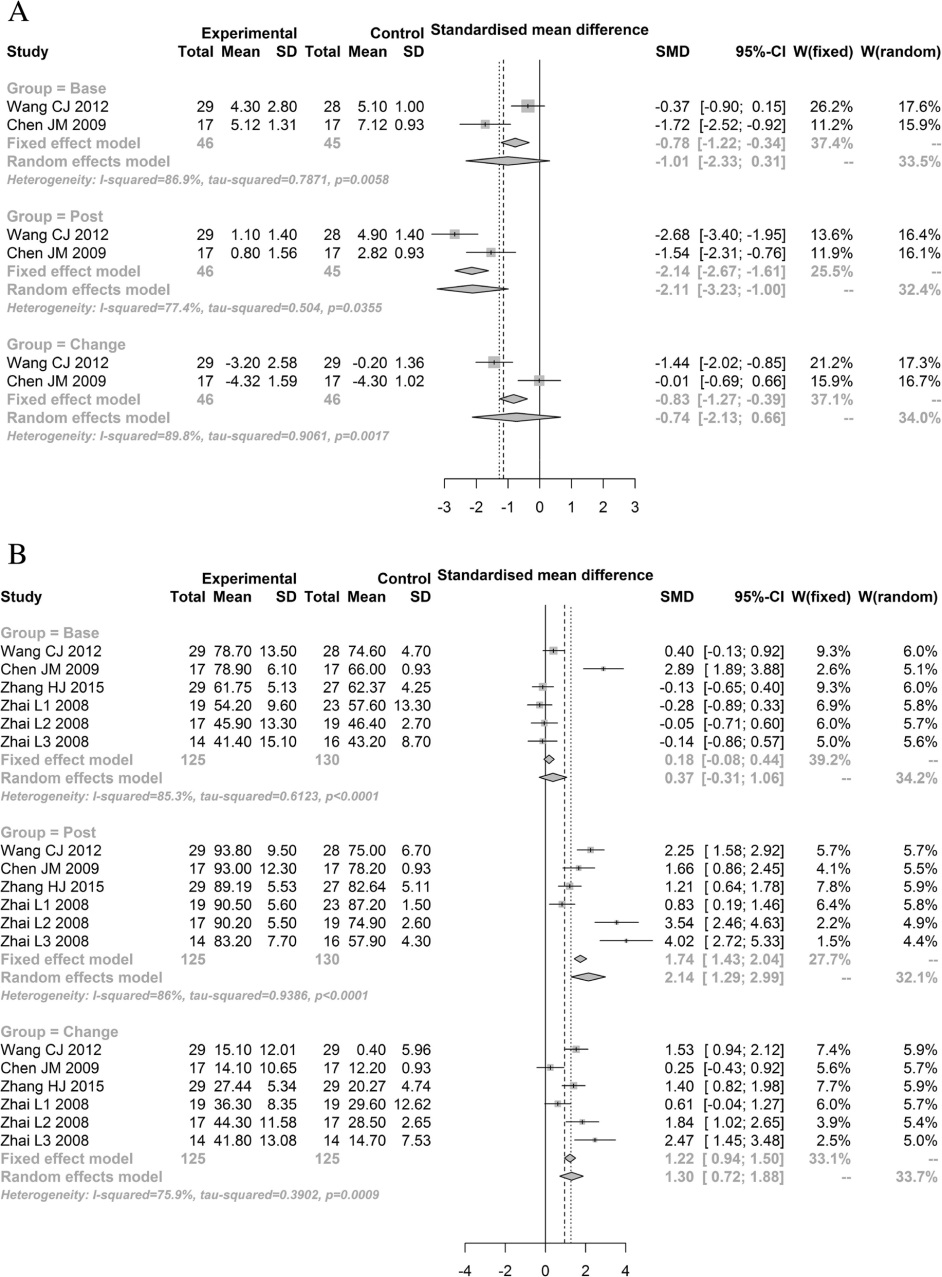
Meta-analysis of pain score and Harris hip score. **a** Pain score. **b** Harris hip score

The pain and Harris hip scores before and after treatment in the experimental and control groups were analyzed. The main results are shown in Table 2 and Fig. 3. No significant differences were found between the two groups in the baseline pain score (SMD = − 1.0104;95% CI − 2.3279–0.3071) and baseline Harris hip score (SMD = 0.3717; 95% CI − 0.3125–1.0559). After a period of treatment, significant differences were found between the experimental and control groups in the pain score (SMD = − 2.1148; 95% CI − 3.2332–0.9965) and Harris hip score (SMD = 2.1377; 95% CI 1.2875–2.9880). No significant differences in pain score were found before and after treatment (SMD = − 0.7353; 95% CI − 2.1272–0.6566), but significant differences were found in the Harris hip score (SMD = 1.2969; 95% CI 0.7171–1.8767).

**Table 2:** Meta-analysis results for pain score and Harris hip score

Variable	Group	Sample size			Test of association			Model	Test of heterogeneity^a, b^			Egger’s test^c^	
		*K*	ESWT	Control	SMD (95% CI)	Z	*P*		Q	*P*	*I*^2^ (%)	*t*	*P*
Pain score	Base	2	46	45	−1.0104 [− 2.3279; 0.3071]	1.5032	0.1328	Random	6.61	0.0058	86.9	–	–
	Post	2	46	45	− 2.1148 [− 3.2332; − 0.9965]	3.7063	0.0002	Random	4.42	0.0035	77.4	–	–
	Change	2	46	46	− 0.7353 [− 2.1272; 0.6566]	1.0354	0.3005	Random	9.81	0.0017	89.8	–	–
Harris hip score	Base	4	125	130	0.3717 [− 0.3125; 1.0559]	1.0647	0.287	Random	34.06	< 0.001	85.3	1.9243	0.1267
	Post	4	125	130	2.1377 [1.2875; 2.9880]	4.9281	< 0.001	Random	35.77	< 0.001	86.0	3.5824	0.0231
	Change	4	125	125	1.2969 [0.7171; 1.8767]	4.3839	< 0.001	Random	20.72	0.001	75.9	0.9755	0.3846

*OR* odds ratio, *CI* confidence interval, *K* number of studies combined

^a^Random-effects model was used when the *P* for heterogeneity test < 0.05; otherwise, the fixed-effect model was used

^b^*P* < 0.05 is considered statistically significant for Q statistics

^c^Egger’s test to evaluate publication bias, *P* < 0.05 is considered statistically significant

### Publication bias

Significant bias was found among individual studies in the comparison of Harris hip scores (*t* = 3.5824, *P* = 0.0231), but no publication bias was found in the change before and after treatment (*t* = 0.9755, *P* = 0.3846) in the baseline pain score and Harris hip score (*t* = 1.9243, *P* = 0.1267).

### Sensitivity analysis

Sensitivity analysis of pain scores demonstrated that the results were unstable, but sensitivity analysis of Harris hip scores demonstrated that the results were stable.

## Discussion

In the present study, we evaluated the role of ESWT in improving ONFH. A total of 230 patients with ONFH were included in the study. No significant difference was found between the two groups in the pain score and Harris hip score before treatment. After treatment, significant differences were found between the experimental and control groups in the pain score and Harris hip score. No significant differences in pain score were found before and after treatment, but significant differences were found in the Harris hip score.

Physical therapy can improve bone oxygenation, reduce edema, reduce bone pressure, improve bone circulation, prevent ischemia, restore blood supply in hypoxic tissue, and promote necrotic bone repair [16]. Wang et al. demonstrated greater improvement with ESWT compared with core decompression and nonvascularized fibular grafting in patients with early-stage ONFH [17]. A previous study demonstrated that ESWT could increase the expression of angiogenic factors, reduce vessel wall stenosis, and improve limb perfusion [18]. The effect of ESWT might be related to stress-induced piezoelectricity, cavitation and osteogenesis, and metabolic activation. These effects promote healing of femoral head necrosis by inducing improved blood circulation, mitigating a hypercoagulable state, and enhancing osteoblast and blood vessel activity [18, 19]. Similar to previous reports, the present study suggested that ESWT might be a safe and effective method to improve motor function and relieve pain, especially at an early stage of ONFH. Significant heterogeneity was observed in the study. Heterogeneity might be introduced by different combined treatment strategies. For example, patients in experimental groups underwent ESWT in two studies [12], while patients in experimental groups in two other studies underwent combined treatment [15, 20]. Although the age difference between groups in individual studies was not significant, the age in the four studies ranged from 20.9 to 40.9 years. Bone density, structure, and strength are correlated with age [21, 22]. Thus, efficacy should be confirmed with further studies after adjusting for background factors that can affect ESWT treatment.

Some limitations should be noted. First, the small sample size introduced more obvious heterogeneity between individual studies [23]. Additionally, the included populations were small and the baseline characteristics of included studies were not complete. Thus, subgroup analysis based on age and sex distribution could not be performed. Second, the quality of the included studies was poor, limiting the strength of the conclusion. Third, publication bias for the Harris hip score after treatment might affect the results. Fourth, only two studies reported pain scores, and further research with larger sample sizes is needed to validate the conclusions.

## Conclusion

For patients at an early stage, ESWT may be a safe and effective way to relieve pain and improve motor function. Nevertheless, due to the low quality of the included publications, the conclusion should be confirmed with further research using a larger sample size. The long-term follow-up studies are favorable to the use of ESWT in ONFH in future.

## Abbreviations

CI: Confidence interval
ESWT: Extracorporeal shockwave therapy
ONFH: Osteonecrosis of the femoral head
SMD: Standardized mean difference
